# Gender Differences in Response to Prolonged Every-Other-Day Feeding on the Proliferation and Apoptosis of Hepatocytes in Mice

**DOI:** 10.3390/nu8030176

**Published:** 2016-03-19

**Authors:** Katarzyna Piotrowska, Maciej Tarnowski, Katarzyna Zgutka, Andrzej Pawlik

**Affiliations:** Katarzyna Piotrowska, Department of Physiology Pomeranian Madical University in Szczecin, al. Powstańców, Wielkopolskich 72, 70-111 Szczecin, Poland; maciej.tarnowski@pum.edu.pl (M.T.); katarzyna.zgutka@pum.edu.pl (K.Z.); pawand@poczta.onet.pl (A.P.)

**Keywords:** caloric restriction, every-other-day feeding, proto-oncogenes, liver, gender differences

## Abstract

Intermittent fasting decreases glucose and insulin levels and increases insulin sensitivity and lifespan. Decreased food intake influences the liver. Previous studies have shown gender differences in response to various types of caloric restriction, including every-other-day (EOD) feeding, in humans and rodents. Our goal was to show the influence of prolonged EOD feeding on the morphology, proliferation and apoptosis of livers from male and female mice. After nine months of an EOD diet, the livers from male and female mice were collected. We examined their morphology on histological slides using the Hematoxilin and Eosine (H_E) method and Hoechst staining of cell nuclei to evaluate the nuclear area of hepatocytes. We also evaluated the expression of mRNA for proto-oncogens, pro-survival proteins and apoptotic markers using Real Time Polimerase Chain Reaction (PCR). We noted increased lipid content in the livers of EOD fed female mice. EOD feeding lead to a decrease of proliferation and apoptosis in the livers of female and male mice, which suggest that tissue maintenance occurred during EOD feeding. Our experiment revealed sex-specific expression of mRNA for proto-oncogenes and pro-survival and pro-apoptotic genes in mice as well as sex-specific responses to the EOD treatment.

## 1. Introduction

Reduced insulin levels, increased insulin sensitivity and low Insulin-like Growth Factor I (IGF-I) levels improves the lifespan of humans and a variety of animal species [1,2,3]. There are several models of increased lifespan. Some findings are based on experiments involving mutations in the GH-IGF-1-insulin axis or pharmacological interventions. Caloric restriction is the only known physiological intervention for improving lifespan [4,5,6,7].

Decreases in food intake may be achieved using different feeding regimes. Some regimes reduce calorie intake every day. This caloric restriction may vary from a 10% reduction, compared to an *ad libitum* (AL) diet, up to 50% [8,9,10]. Another type of dietary intervention is every-other-day feeding (EOD), also known as intermittent fasting (IF). In this method the organism is deprived of food for 20–24 h, which is preceded by AL feeding [11,12,13].

Intermittent fasting positively affects lifespan, glucose levels, glucose tolerance, insulin levels and insulin sensitivity in rodents and humans [13,14]. It also helps to reduce weight in rodents and humans [13,14,15]. Even short-term fasting influences the body’s greatest metabolic center, the liver. Short-term experiments of fasting and refeeding in rodents revealed changes in liver weight, glycogen levels, proliferative index and the number of cells undergoing apoptosis [16,17]. Longer experiments (one up to four months of intermittent fasting) revealed decreases in reactive oxygen species (ROS) production and changes in the activity of antioxidative and carbohydrate metabolizing enzymes in rodent models of fasting [12,18].

There are also data indicating gender differences in response to caloric restriction, or pharmacological treatment mimicking caloric restriction (CR) action [19,20,21]. In a previous study, we also noted differences in response to EOD among female and male mice [22]. We observed loss of weight in males but no differences in weight among females. Long-term EOD also influenced the morphology of the ovaries, with a larger pool of primordial, primary and early secondary follicles seen in the ovaries of mice undergoing intermittent fasting [22]. However, we found no histological changes in the testes of male mice.

In the present study, our goal is to investigate the effects of long-term (up to nine months) EOD on the morphology, proliferation and apoptosis of the livers of C57BL/6 male and female mice.

## 2. Material and Methods

### 2.1. Animals

A total of 24 four-week-old C57Bl/6 mice of both sexes were employed in our experiments. Mice were randomly divided into two groups (6 animals/group of each sex) and were housed separately in isolated cages (one mouse per cage) under controlled conditions of optimum temperature (22 °C), Labofeed H (containing 60% carbohydrates, 30% proteins and 10% fat) (Morawski, Poland) *ad libitum (*AL), and the other half of the animals were placed on every other day (EOD) feeding regiment. EOD feeding regimen involved alternating *ad libitum* feeding with Labofeed H and fasting every other day food [13]. Food intake was measured at 5 p.m. every day, when food was given or removed. Body weight was measured once a week starting on Day 0. At the end of the study (after 9 months), the mice were sacrificed. During necropsy, livers were isolated, weighed and prepared for further analysis. All animal protocols were approved by the Local Ethical Committee (approval number 27/2012).

### 2.2. Tissue Preparation

Livers for histological and immunohistochemical analysis were fixed in 10%-buffered formalin for 24 h, and after fixation, samples were dehydrated and embedded in paraffin blocks. For RQ-PCR livers were immediately frozen in liquid nitrogen.

### 2.3. Histological Analysis

Deparaffinized sections of livers (3 μm thick) were rehydrated and stained with Mayer’s hematoxylin and eosin (H&E) stain according to standard procedures. After staining, sections were dehydrated in 95% and 99.8% alcohol, cleared with xylene mounted with Canada balsam (all purchased in Sigma-Aldrich, Saint Louis, MI, USA) mounting medium, and evaluated under an Olympus IX81 inverted microscope (Olympus, Germany). Micrographs were collected with CellSens software (Olympus).

### 2.4. Immunohistochemical Analysis of Proliferative and Apoptotic Markers

Deparaffinized sections of livers (3 μm thick) were hydrated and heat epitope retrieval was performed in microwave oven in retrieval solution buffer pH = 6. After cooling to room temperature (RT), the slides were incubated with 0.3% solution of H_2_O_2_, washed twice with PBS and further incubated with 2.5% horse serum. After washing in PBS slides were incubated with primary antibody: rabbit anti-mouse Ki67 (Novus, USA) and rabbit anti-mouse Bax (Santa Cruz Biotech., USA) for 1 h in RT. After washing in PBS immunoreaction was visualized with ImmPRESS UNIVERSAL REAGENT and Vector NovaRED Substrate KIT FOR PEROXIDASE (VECTOR LABORATORIES, USA) according to manufacturer protocol. As a negative control, the primary antibody was replaced with PBS on the specimen. Positive staining was defined by visual identification of a yellow/brown pigmentation in the light microscope. Images were collected with an Olympus IX81 inverted microscope (Olympus) with color camera and with CellSens image processing software (Olympus).

### 2.5. Hepatocytes Nuclear Size and Nuclear Area

Nuclear size of hepatocytes was determinated by the method described elsewhere [23]. Briefly, on H&E slides 100 hepatocytes’ nuclei per animal were measured for their major (a) and minor (b) axes. The hepatocytes nuclear area (S) was calculated as S = Πab/4. Hepatocyte nuclear area was expressed as average ± SD and median.

Hepatocytes’ heterogeneity was assessed by staining liver slides with Hoechst 34580 for 30 min in room temperature (RT), and after double wash in PBS slides were mounted in fluorescent mounting medium.

### 2.6. Real-Time Quantitative Reverse Transcription PCR (RQ-PCR)

Total RNA was isolated from liver samples of experimental (EOD) and control (AL) mice with the RNeasy Kit. RNA (Qiagen, Hilden, Germany) was reverse-transcribed with First Strand cDNA Synthesis Kit (Thermo Scientific, Waltham, MA, USA). Quantitative assessment of mRNA levels was performed by real-time reverse transcriptase polymerase chain reaction (RT-PCR) on an ABI 7500 Fast instrument using Power SYBR Green PCR Master Mix reagent (Applied Biosystems, USA). Real-time conditions were as follows: 95 °C (15 s), 40 cycles at 95 °C (15 s), and 60 °C (1 min). According to melting point analysis, only one PCR product was amplified under these conditions. The relative quantization value of target (the fold change) normalized to the endogenous control β-2 microglobulin gene and relative to a calibrator is expressed as 2^∆∆Ct^, where ∆Ct = (Ct of target genes) − (Ct of endogenous control gene, β-2 microglobulin), and ∆∆Ct = (∆Ct of sample for target gene) − (∆Ct of calibrator for the target gene). Sequences of primers used are presented in Table 1.

### 2.7. Statistical Analysis

All results are presented as average ± SD and median. Statistical analysis of the data was done using Student’s t-test for unpaired samples, with *p* < 0.05 considered significant. In comparison of expression of mRNA for given gene between the control females and males change lower than 3-fold was consider insignificant.

### 2.8. Statistical Analysis

All results are presented as means ± SD and medians. Statistical analysis of the data was done using a Student’s t-test for unpaired samples, with *p* < 0.05 considered significant. Changes in the expression of mRNA for given genes lower than three-fold were considered insignificant.

## 3. Results

### 3.1. Body and Liver Mass

All animals increased in body weight. The gain was significantly lower in animals fed the EOD diet (45% *vs.* 21% and 41% *vs.* 31% for males and females, respectively). The EOD diet was more effective for males, as their body weight increased less than females (21% *vs.* 31%). These results are summarized in Table 2. Cumulative food intake per animal during experiment was greater in AL animals than EOD (AL Females 746.85 ± 13.79 g *vs.* EOD females 657.97 ± 1.76 g; AL males 949.65 ± 8.27 g *vs.* EOD males 698.26 ± 4.94 g per animal). The diet caused decreases in liver mass among EOD male mice compared to AL fed animals, but this result was not statistically significant (Table 3). Among females, EOD caused increases in liver mass when compared to AL fed females, but this result was also not statistically significant (Table 3).

### 3.2. Histology of the Liver

**Figure 1:**
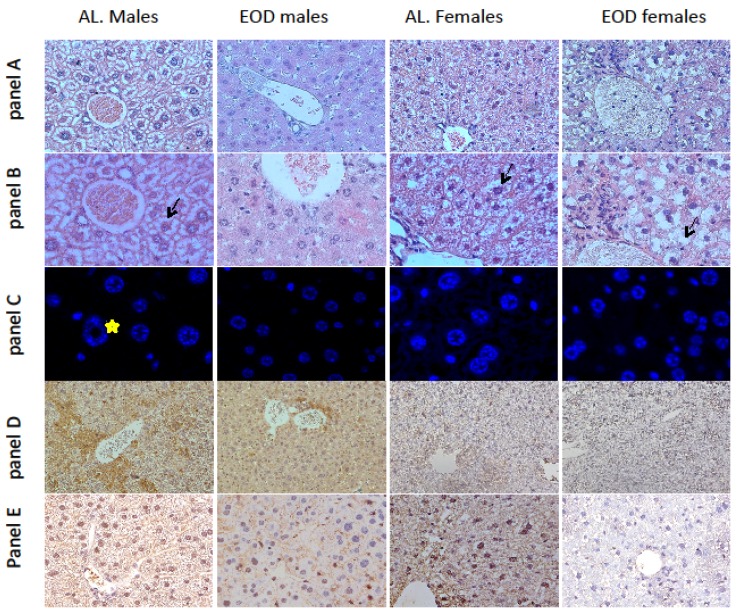
Morphology of liver sections of male and female mice on EOD diet and AL fed. Representative microphotographs of livers are presented. Panel (**A**,**B**)—morphology of liver tissue, H-E staining, magnification of panels: (**A**) 400×, (**B**) 600× (oil immersion); black arrows indicate lipid vacuoles in hepatocytes of AL fed males, AL and EOD fed females; Panel (**C**)—Hepatocytes’ nuclei heterogeneity, Hoechst 34580 staining, magnification of panel (**C**) 400×; yellow asterisk indicates vacuole in hepatocyte’s nucleus of AL fed male mice; Panel (**D**)—Immunostaining of Bax in liver tissue; Yellow/brown pigmentation indicates positive immunoreactions in cytoplasm nuclei of cells, magnification of panel (**D**) 400×; Panel (**E**)—Immunostaining of Ki67 in liver tissue; Yellow/brown pigmentation indicates positive immunoreactions in nuclei of the cells, magnification of panel (**E**) 400×.

In the histological sections of EOD males we noted a small amount of small-size lipid droplets. The hepatocyte plates were well developed and sinusoids were clearly visible. We observed no signs of inflammation (Figure 1A,B). In the H-E sections of AL fed males, we observed significant deposits of lipids in the cytoplasm of the hepatocytes indicated with black arrow on panel B AL Males. In addition, the hepatocytes and sinusoids were enlarged and the plates of the hepatocytes were well developed. We observed no signs of inflammation (Figure 1A,B). Sections of liver collected from EOD female mice revealed large lipid droplets in the hepatocytes as compared to AL fed females indicated with black arrow on panel B ALl Females and EOD Females. In addition, the hepatocyte plates were well developed and the sinusoids were poorly visible compared to AL fed females.

### 3.3. The Nuclear Area of Hepatocytes

A nuclear area and heterogeneity of hepatocytes are known markers of aging in liver tissue [24]. We compared the nuclear area of hepatocytes from all experimental groups of mice. We found significantly smaller nuclear areas in males fed EOD compared to AL fed males (Table 4). In addition, the nuclear area of EOD fed females was significantly smaller than in AL fed females (Table 4). Our measurements also indicated differences in the nuclear area of AL fed males and females. In animals without restrictions, the nuclear area of hepatocytes among females was smaller than among males (Table 4). We also noted an increase in the heterogeneity of hepatocytes among AL male and female mice compared to their EOD fed littermates (Figure 1C). In liver sections of AL fed males we revealed the presence of nuclei containing vacuoles indicated with yellow asterisk on Figure 1 panel C.

### 3.4. Immunolocalization of Ki67 and Bax

We also established immunolocalization for the proliferative marker Ki67 and apoptotic marker Bax in the liver tissue slides (Figure 1D,E). We observed decreased immunoreactivity for Ki67 and decreased immunoexpression for Bax in EOD males and females compared to controls. These results are in line with our data on mRNA expression of proliferation and apoptosis markers.

### 3.5. Expression of c-jun

**Figure 2:**
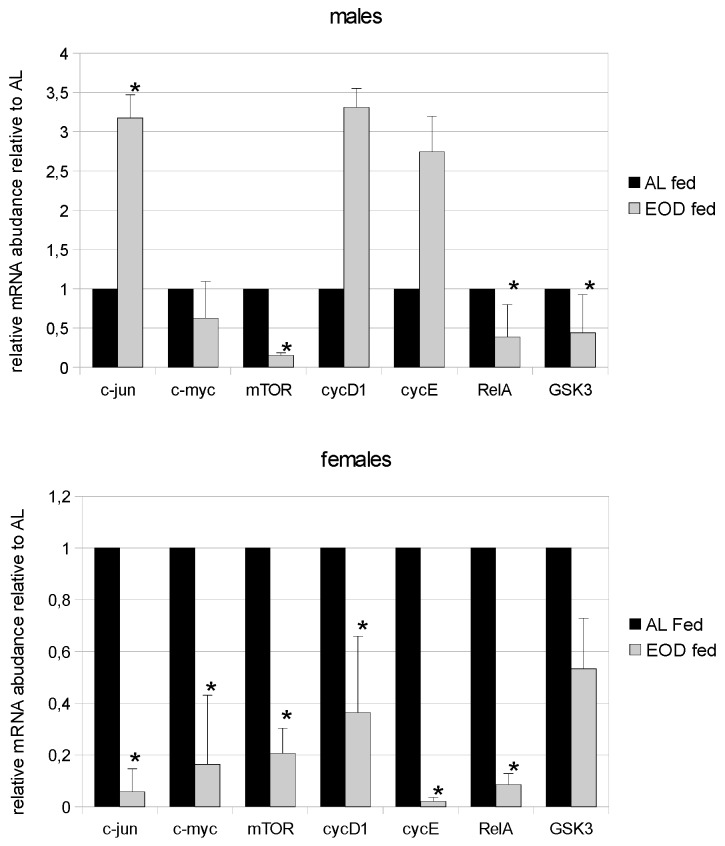
Expression of mRNA for chosen protooncogenes in male and female mice on EOD diet and AL fed. Data expressed as relative mRNA abundance—fold change relative to AL animals as 1. Significantly different from AL group *p < 0.01.

An increased expression of c-jun has been found in hepatocellular carcinomas [25]. In our study, we observed a significant increase in the levels of c-jun in EOD fed male mice compared to AL fed males and a significant decrease in the expression of c-jun in EOD females compared to control females (Figure 2). We also noted that among control animals, levels of c-jun expression were almost 55 times higher in females than in males (Figure 2).

### 3.6. Expression of the c-myc Proto-Oncogene

Previous studies have shown that the expression of c-myc plays a significant role in liver malignancies [25]. The quantitative Real Time PCR revealed increased expression of c-myc mRNA in the hepatocytes of AL fed male mice compared to EOD fed males; however this result was not statistically significant (Figure 2). Among EOD fed females, we observed a significant decrease in the expression of c-myc mRNA compared to AL fed females (Figure 2). Interestingly, in control animals, the level of expression of c-myc mRNA in females was 3.8 times higher than in males.

### 3.7. Expression of mTOR

Mammalian (Mechanistic) Target Of Rapamycin (mTOR) is a protein kinase, sensitive to nutritional status, that up-regulates protein and lipid synthesis, is required for cell proliferation, down-regulates autophagy and lysosome biogenesis and is responsible for cell survival [26]. In our experiment, we observed a significant decrease in the expression of mRNA for mTOR among EOD males and females compared to AL males and females (Figure 2). We also observed a higher expression of mRNA for mTOR in control males compared to females; however, this difference was not statistically significant.

### 3.8. Expression of Cyclin-D (cycD) and Cyclin-E (cycE)

Cyclins are proteins responsible for the progression of the cell cycle. CycD and cycE are used as markers of regeneration and also tumor progression [27,28,29]. In this study, we noted a slight increase in the expression of cycD and cycE among EOD fed males compared to AL fed males; however, among EOD females cycD and cycE expression was significantly decreased compared to AL fed females (Figure 2). Additionally, we observed gender specific differences in the expression of mRNA for cycD and cycE in control animals, with higher levels among AL females than AL males.

### 3.9. Expression of RelA (p65)

RelA (p65) is one of the NF-κB proteins responsible for inflammation and carcinogenesis, as well as the regulation of the expression of genes related to inflammation and apoptosis [30]. In our study, we found a decrease in the mRNA expression for the RelA (p65) gene in EOD females and males compared to controls (Figure 2). We also observed higher levels of mRNA for RelA in the livers of AL females compared to AL males.

### 3.10. Expression of GSK3

Glycogen Synthase Kinase 3 (GSK3) is the kinase involved in glycogen metabolism, cell differentiation, proliferation and survival [31]. It participates in signaling pathways for systems that are often deregulated in liver cancers [32]. In our study, AL fed males displayed significantly higher mRNA expression of GSK3 in their livers compared to their EOD fed littermates (Figure 2). Among females on the EOD diet, we noted a decrease in the levels of mRNA for GSK3 compared to AL females; however, these differences were not statistically significant. Additionally, we observed no significant changes in the expression of mRNA for GSK3 between control females and males.

### 3.11. Expression of Bcl-2 and Bcl-x_L_

**Figure 3:**
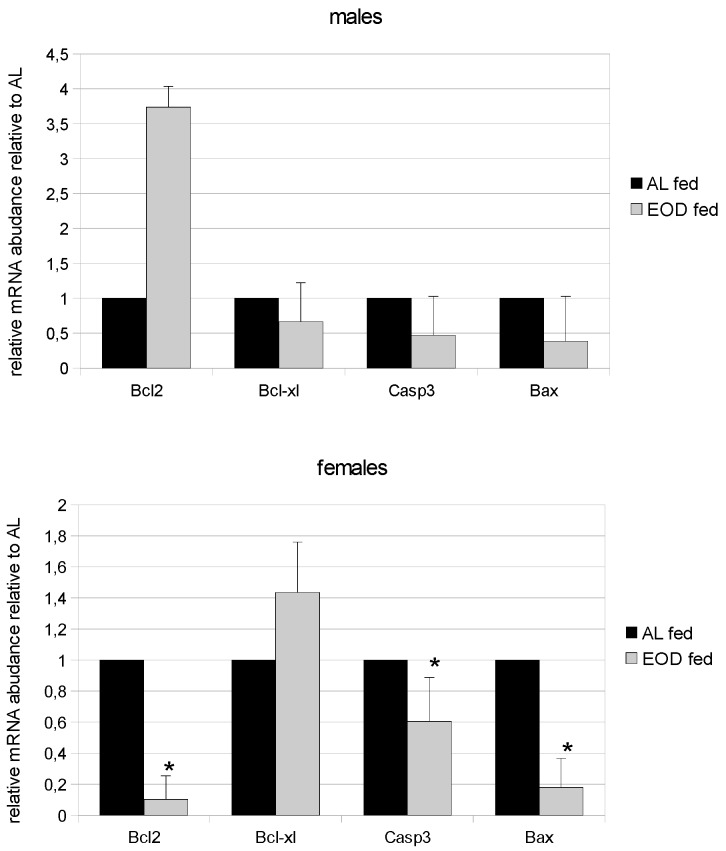
Expression of mRNA for chosen proapoptotic and antiapoptotic proteins in male and female mice on EOD diet and AL fed. Data expressed as relative mRNA abundance—fold change relative to control (AL). Significantly different from AL group * *p* < 0.01.

Bcl-2 is a pro-survival protein and is required for the survival of mature lymphocytes, melanocyte stem cells and cells developing in the kidney. Bcl-x_L_ is required for proper embryonic development, as embryos lacking Bcl-xL die in utero [33]. In our study, mRNA expression for the Bcl-2 protein was significantly increased among EOD males and significantly decreased among EOD females compared to controls (Figure 3). We also found a difference in the expression of Bc1-2 mRNA between control females and males. In males, we observed higher levels of Bcl-2 mRNA than among females. The expression of Bcl-xL mRNA was significantly decreased among EOD fed males compared to AL males and significantly increased among EOD females compared to AL fed females. Moreover, we observed no significant changes in the expression of mRNA for Bcl-xL between control females and males.

### 3.12. Expression of Caspase 3 and Bax

The expression of apoptotic proteins (caspase 3 and Bax) indicates apoptosis. In EOD animals (both males and females) we observed a decrease in mRNA expression for caspase 3 and Bax compared to AL males and females (Figure 3); however, these differences were only statistically significant for females. We observed no significant changes in the expression of mRNA for caspase 3 and Bax among control females and males.

## 4. Discussion

It has been established that a reduction in calorie intake via CR, EOD or Ramadan type fasting leads to increased life span, decreased levels of GH-IGF-1 and insulin and increased sensitivity to insulin in humans and animals [9,13,34,35,36,37]. After long-term EOD treatment in mice, we observed lower increases in body mass among males, as well as changes in liver weight and morphology among males and females. A reduction in body mass was one the first results highlighted from food restriction and has been found in many previous experiments using CR and EOD [34,35,36,37,38]. There are only a few studies that have observed a lack of a decrease in body weight among experimental animals [11,12]. In the present study, we observed lower increases in body weight among males but no changes in the increase of body weight during the experiment among females on EOD diet compared to control mice. Similar body mass among females at the end of experiments has been noted by others using different mice strains and also C57BL/6J [11,12]. We assume that the lack of weight loss may be the result of a disturbed estrogen balance during chronic EOD treatment among female mice as we observed greater food intake in AL females than EOD females measured as cumulative food intake during experiment. It has already been established that food restriction decreases circulating levels of estrogen in female rodents [39]. We have also shown that morphological changes occur in the ovaries during nine-month-long EOD [22], *i.e.*, a sustained pool of early-stage follicles and a small amount of mature follicles. Maturation of ovarian follicles depends on estrogen status among females and may cease during decreased estrogen levels. We also noted an increase in the expression of estrogen receptors, which may indicate decreased circulating level of this hormone and increased susceptibility to its action [40]. Apart from their gonadal function, estrogens exert a strong influence on other tissues, such as the liver, adipose tissues and skeletal muscles (reviewed in [41]). In the liver, estrogens are responsible for lipid homeostasis and marked steatosis has been noted in the studies, which report decreased estrogen levels [42,43]. Stubbins *et al.* (2012) showed hepatosteatosis among ovariectomized mice and a lack of hepatosteatosis among ovariectomized mice supplemented with estrogen and among non-ovariectomized mice [44]. In our study, we also observed marked steatosis in the livers of EOD females but not in males. During prolonged conditions of decreased calorie intake, the metabolism of female livers shifted toward lipid storage, observed as steatosis [45].

A nuclear area and heterogeneity of the nuclei of hepatocytes are markers of an aging liver that are also found during accelerated aging in mice and mutants that overexpress bovine GH [24,31]. In our current study, we observed a decreased nuclear area and lower levels of heterogeneity in hepatocytes among EOD animals, which may indicate decreased levels of GH/IGF-1 signaling and a younger biological age for this organ among EOD fed mice. The presence of nuclei containing vacuoles is also a symptom of aging [24] and presence of this change in AL fed male mice and lack of vacuolated nuclei in EOD fed males may suggest younger biological age for this organ among EOD fed mice.

In our study, we evaluated mRNA levels for proto-oncogenes. Some of these genes are involved in regeneration and survival, while others are known as markers of tumour genesis, mainly in the liver [45,46,47,48]. CR and EOD influence lifespan by decreasing carcinogenesis (reviewed in [49]). Decreases in the expression of c-jun, c-myc, mTOR, cycD1, cycE and RelA among females and mTOR, RelA, GSK3 among males during chronic EOD treatment shows the beneficial aspects of this intervention and partly explains the observed prolongation of lifespan in animals and humans during decreased food intake treatments [50]. An increase of proto-oncogene expression in mRNA and protein levels has also been shown in mice overexpressing bovine GH and with high levels of IGF-1 [31]. This indicates a strong connection between proto-oncogene expression and IGF-1 levels. It supports the notion that decreases in GH-IGF-1 trigger shifts in energy usage towards tissue maintenance instead of proliferation [51].

We also observed that there were statistical differences in the expression of c-jun, c-myc, cycD1 and cycE mRNAs between AL fed (control) males and females. This suggests gender-specific expression of these genes in the liver of C57BL/6 mice. A larger decrease in mRNA for proto-oncogenes was observed in females. This is consistent with the influence of estrogens on c-myc, cycD1 and cycE, which are up-regulated by estradiol, as already confirmed in adipose tissue, some cancers and hepatocytes [52,53,54,55]. The down-regulation of proto-oncogenes observed in our experiment may be explained by a decrease in estradiol levels among EOD fed mice. Gender differences in gene expression in the liver have already been confirmed for drug and steroid metabolizing genes [56,57,58] and also the reduced glutathione (GSH) antioxidant system [59]. A higher basic expression of c-jun was also found in cardiac fibroblasts from male rats compared to females, while cycD1 expression was comparable in both genders [60].

mRNA expression for survival proteins (Bcl-2 and Bcl-_XL_) showed an elevation in the mRNA expression of Bcl-2 in EOD treated male mice. This may indicate a “pro-survival” state in liver tissue under conditions of decreased food intake. We also noted a significant difference in the basic expression of mRNA for this protein, which may indicate a gender-specific expression for this pro-survival marker. This has already been described in rat cardiomyocytes and in the medial preoptic nucleus, with higher expression of Bcl-2 found in males compared to females [61,62]. We noted a decrease in Bcl-2 mRNA expression in EOD females that could be the result of decreased estrogen levels. Wang *et al.* 2011 showed that estradiol levels positively correlate with Bcl-2 expression in cultured neurons and that decreases in estradiol triggers decreases in the expression of Bcl-2 [63].

The restriction of food intake leads to changes in levels of apoptosis. It has been postulated that increased level of apoptosis in tissues eliminates damaged cells and, on the scale of multicellular organisms, helps to maintain health [64]. In previous studies using CR, levels of apoptosis in the liver increased and correlated with the liver neoplasmic incidence in mice [65]. There are also reports indicating decreased levels of caspase 3 and Bax during food restriction in kidneys [66] and decreases of Bax in the livers homogenates of male C57Bl/6J mice fed on a CR soybean oil diet [67]. In mutant mice, with undetectable IGF-1 level (GHR-KO), the level of caspase 3 and Bax was found to decrease in skeletal muscles, while levels of bcl-2 increased. However, CR does not change the level of those markers [68]. In our study, we also observed significant decreases in the expression of mRNA for caspase 3 and Bax in females but not males. All experiments with caloric restriction, regardless of model, are long-lasting feeding regimes with a 40% decrease in calorie intake. Muskhelishvilli *et al.* 1995 showed apoptotic bodies, DNA fragmentations in liver tissue, while other teams focused on the expression of pro-apoptotic proteins [65,66,67,68]. Our results on the expression of pro-apoptotic proteins, combined with results concerning anti-apoptotic markers and proto-oncogenes, suggest that prolonged EOD feeding promotes cellular maintenance in the liver and is more pronounced in females than males.

In conclusion, a chronic every-other-day feeding regime influences liver morphology, weight and mRNA levels for proto-oncogenes, as well as pro-survival and pro-apoptotic proteins. The response to food restriction is sex-specific and promotes tissue survival and reduced cellular turnover in the liver.

## Figures and Tables

**Table 1:** The sequences of the primers used to real-time PCR.

c-myc-forward	5′-TCACCAGCACAACTACGCCG
c-myc-reverse	5′-TGCTTCAGGACCCTGCCACT
c-jun-forward	5′-CATTGCCGCCTCCAAGTG
c-jun-reverse	5′-CCAGCTCGGAGTTTTGCG
mTOR-forward	5′-GTTTGTGGCTCTGAATGACC
mTOR-reverse	5′-TCAGGATCTGGATGAGCATC
cyclD1-forward	5′-GCGAAGTGGAGACCATCCG
cyclD1-reverse	5′-GGTCTCCTCCGTCTTGAGC
RelA-forward	5′-GCTCCTGTTCGAGTCTCCAT
RelA-reverse	5′-TAGGTCCTTTTGCGCTTCTC
cyclE-forward	5′-GGCGGACACAGCTTCGGGTC
cyclE-reverse	5′-TGGGTCTTGCAAAAACACGGCCA
GSK3b-forvard	5′-CCACCATCCTTATCCCTCCAC
GSK3b-reverse	5′-GTATCTGAGGCTGCTGTGGC
Bcl-2-forward	5′-GTC CCG CCT CTT CAC CTT TCA G
Bcl-2-reverse	5′-GAT TCT GGT GTT TCC CCG TTG G
Bcl-xL -forward	5′-AAC ATC CCA GCT TCA CAT AAC CCC
Bcl-xL-reverse	5′-GCG ACC CCA GTT TAC TCC ATC C
Bax -forward	5′-GCG TGG TTG CCC TCT TCT ACT TTG
Bax-reverse	5′-AGT CCA GTG TCC AGC CCA TGA TG
Casp3-forward	5′-ATGGAGAACAACAAAACCTCAGT
Casp3-reverse	5′-TTGCTCCCATGTATGGTCTTTAC

**Table 2:** Body weight of animals on every-other-day (EOD) diet and *ad libitum* (AL) fed during 9 months of treatment.

Body Weight (g)	Beginning of the Experiment	End of the Experiment	Gain of Weight (%)
**AL males**	28.66 ± 0.75	41.79 ± 4.96 *	45.81%
**EOD. males**	24.48 ± 1.47	29.67 ± 2.54 *	21.2%
**AL. Females**	21.24 ± 1.15	30.13 ± 3.35	41.85%
**EOD Females**	22.14 ± 1.97	29.13 ± 2.46	31.57%

Significantly different from AL group * *p* < 0.05.

**Table 3:** Body mass and liver mass of animals on EOD diet and AL fed during 9 months of treatment.

	Body Weight (g)	Liver Weight (g)	Liver/Body Weight
**AL. Males**	41.79 ± 4.96 *	1.74 ± 0.44	4.16%
**EOD Males**	29.67 ± 2.54 *	1.42 ± 0.14	4.78%
**AL. Females**	30.13 ± 3.35	1.30 ± 0.37	4.31%
**EOD Females**	29.13 ± 2.46	1.42 ± 0.17	4.87%

Significantly different from AL group * *p* < 0.05.

**Table 4:** Hepatocyte nuclear area (µm^2^) of animals on EOD diet and AL fed.

	AL Males	EOD Males	AL Females	EOD Females
Average	72.66 *	51.02 *	54.02 *	32.63 *
SD	±25.51	±14.97	9.81	8.45
median	70.10	48.60	54.11	30.30

Significantly different from AL group * *p* < 0.001.
